# Attitudes of Children, Adolescents, and Their Parents Toward Digital Health Interventions: Scoping Review

**DOI:** 10.2196/43102

**Published:** 2023-05-02

**Authors:** Arnaud d'Halluin, Marie Costa, Margot Morgiève, Déborah Sebbane

**Affiliations:** 1 WHO Collaborating Centre for Research and Training in Mental Health EPSM Lille Métropole Lille - Hellemmes France; 2 Évaluation clinique épidémiologique-économique appliquée aux populations vulnérables Inserm Université Paris Cité Paris France; 3 Centre de recherche médecine, sciences, santé, santé mentale, société Inserm Université Paris Cité Paris France

**Keywords:** eHealth, mental health, children and adolescents, attitude, scoping review, mobile phone, digital health intervention, DHI

## Abstract

**Background:**

The prevalence of mental health problems in children and adolescents is high. As these problems can impact this population’s developmental trajectories, they constitute a public health concern. This situation is accentuated by the fact that children and adolescents infrequently seek help. Digital health interventions (DHIs) offer an opportunity to bridge the treatment gap between health care needs and patient engagement in care. Additional detailed research is needed to identify how children and adolescents can be empowered to access help through DHIs. In this context, an understanding of their attitudes toward digital health appears to be a necessary first step in facilitating the effective implementation of DHIs.

**Objective:**

This study aimed to establish an inventory of children’s, adolescents’, and their parents’ attitudes toward DHIs.

**Methods:**

A scoping review following PRISMA-ScR (Preferred Reporting Items for Systematic reviews and Meta-Analyses extension for Scoping Reviews) recommendations was performed using the MEDLINE, Embase, and PsycINFO databases. This research was conducted using 3 key concepts: “child and adolescent mental health service users,” “digital health interventions,” and “attitudes.” Data extracted included the name of the publishing journal, the methodology used, the target population, the DHI studied, and the principal results.

**Results:**

Of 1548 studies found, 30 (1.94%) were included in our analysis. Among these, 13 concerned satisfaction, 24 concerned preferences, 22 concerned the use of DHI, 11 concerned perception, and 10 concerned needs.

**Conclusions:**

The results of this study provide a better understanding of the factors influencing children’s and adolescents’ attitudes toward digital health and DHIs. The continued growth of DHIs can help reduce barriers to mental health care. Future research on these interventions should investigate the needs of the targeted populations to increase their engagement in care.

## Introduction

### Background

Mental health problems among children and adolescents can severely affect several areas of their life and developmental trajectories [1]. Worldwide, the estimated prevalence of these problems in this population ranges from 10% to 20% [2]. In 2015, an international meta-analysis found that the prevalence of mental health problems in children and young people was 13.4%, with the most frequent mental health problems being anxiety and disruptive behaviors [3]. Mental health problems among children and adolescents constitute a public health issue, especially because they may be reluctant to seek professional help [4,5]. Reasons for not seeking help include the stigmatization of mental problems, lack of confidentiality and trust, stress associated with seeking help, fear of care providers, and no access to care [5]. The growing interest in technology among children and adolescents may offer a unique opportunity to bridge what has been called the “treatment gap” between mental health care needs and care seeking in this population [6].

### Digital Health Interventions in Mental Health

Digital health interventions (DHIs) comprise web-based interventions, mobile apps, mobile text messages, virtual reality environments, and computer programs. They are easy to access and guarantee anonymity [7]. Several public health organizations have recognized DHIs as a cost-effective, scalable solution to help solve the treatment gap for mental health problems [8]. In 2015, a World Health Organization survey revealed that 29% of the 15,000 health apps surveyed focused on mental health diagnosis, treatment, or support, constituting a disproportionate and noncoordinated offer [9]. Although many of these apps were evidence informed and were inspired by scientifically validated techniques and methods, very few were evidence based (ie, the subject of rigorous scientific studies demonstrating their effectiveness and reliability [10,11]). Most recently, lockdowns related to the COVID-19 health crisis forced mental health services to sharply increase their use of information and communications technology (ICT) to provide care, with a consequent acceleration in DHI implementation [12]. However, despite this growing interest in eHealth, more research is needed to support the development and consequent implementation of DHI [7].

### Research’s State of the Art

Researchers have emphasized several limitations of their implementation, including poor patient engagement, high dropout rates, and interventions that are inadequately tailored to patients’ needs [7,13]. Engagement (also described as participation or adherence, whereas its absence is described as noncompliance or resistance) is commonly referred to as the active involvement of participants in an intervention [14]. Recently, a meta-analysis found that engagement in DHIs by children and adolescents depended on “intervention-specific influences” (suitability, usability, and acceptability) and “person-specific influences” (motivation, opportunity, and the capability to use) [7]. These influences should be considered when providing a DHI [7]. Another systematic and meta-review found that children and adolescents with mental health problems who used DHIs expressed the need for them to be accessible and controllable and that they could be used with autonomy. It also suggested that their attitudes should be taken into account when designing a DHI [15].

Attitude is defined as “an enduring, learned predisposition to behave in a consistent way toward a given class of objects, or a persistent mental and neural state of readiness to react to a certain class of objects, not as they are, but as they are conceived to be” [16]. It manifests both psychologically and sociologically as a subjective predisposition of what a person likes and a prescriptive judgment of what a person ought to do [17]. Attitude can be measured using beliefs, emotions, and behaviors [17]. Previous literature on attitudes toward DHIs found that it can reflect (1) acculturation to ICT and eHealth literacy, (2) trust or mistrust, and (3) resistance or predisposition to its use [18,19]. As attitude contains a priori notions about the DHI, it may directly affect engagement in these interventions.

### Objectives

There is a scarcity of literature on attitudes of young people toward DHIs [18]. In this context, we conducted a scoping review of the attitudes of children and adolescents with distressing mental experiences toward the DHI they had already used. Specifically, we aimed to create an inventory of children’s, adolescents’, and their parents’ attitudes toward DHIs, with the aim of acquiring a better understanding of young DHI users’ attitudes toward this technology and their engagement in it, as well as to guide future research in this field.

## Methods

### Overview

The protocol for this review followed the PRISMA-ScR (Preferred Reporting Items for Systematic reviews and Meta-Analyses extension for Scoping Reviews) extension recommendations for scoping reviews [20]. It was written in June 2021 by the French research team of the World Health Organization Collaborating Centre for Research and Training in Mental Health (Lille, France), which comprises a resident psychiatrist (AD), a researcher in public health (MC), a researcher in social sciences (MM), and a senior psychiatrist (DS). All the authors (AD, MC, MM, and DS) defined the search strategy, the inclusion and exclusion criteria, the selection process, and the data extraction method. The protocol was registered on the Open Science Framework [21].

### Identifying Relevant Studies

The search strategy consisted of identifying key concepts related to DHIs, targeted at children and adolescents, and how they were perceived by users. We developed a provisional syntax for each database to pretest and determine the definitive Boolean equation used. Boolean equations were structured around 3 domains: “digital mental health intervention,” “child or adolescent mental health service user,” and “attitude.” The keywords used came from everyday vocabulary and thesaurus terms.

We used 3 databases, MEDLINE, Embase, and PsycINFO, which cover the fields of medicine, psychology, behavioral science, and DHIs. The results were filtered to retain only articles concerning children and adolescents, using MEDLINE’S age limits for children (0-18 years). The syntax and limits used for each database searched can be found in Multimedia Appendix 1.

### Inclusion and Exclusion Criteria

Studies had to be written in English and published between January 1, 2007 (the year when the first smartphone was released), and July 8, 2021 (the date of the last literature search). The study population in each study included children, adolescents, or their parents. Only studies whose primary objective was to explore DHI use by children or adolescents, their preferences, or their attitudes were included in the analysis. The following types of articles were excluded: (1) articles where the studied DHI only targeted parents of children, (2) articles that did not interview users but focused on the implementation of DHIs or on DHI efficacy studies, and (3) articles that only explored professional mental health care providers’ attitudes toward DHIs.

### Selecting Studies

**Figure 1:**
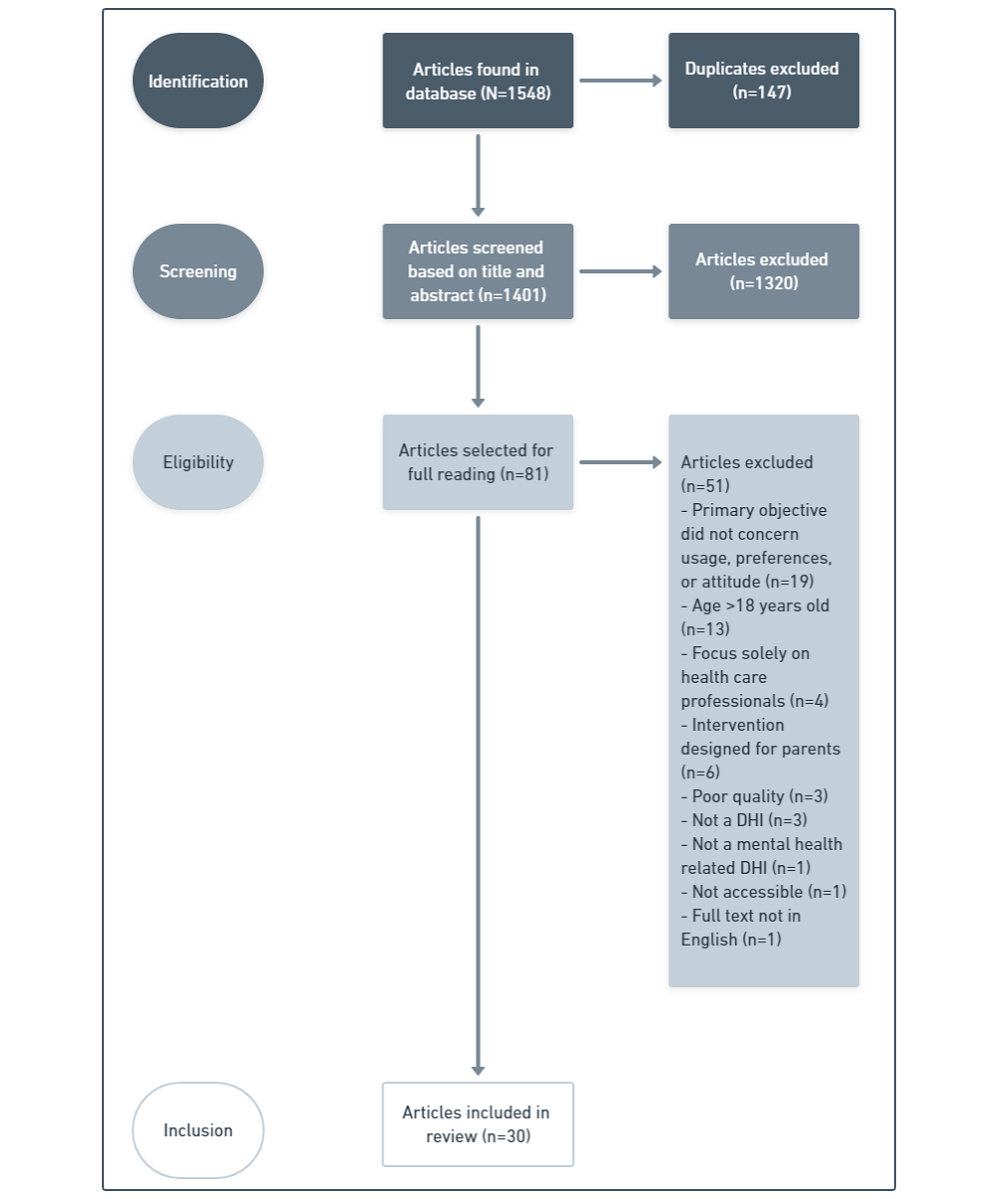
PRISMA-ScR (Preferred Reporting Items for Systematic reviews and Meta-Analyses extension for Scoping Reviews) flowchart. DHI: digital health intervention.

The first author (AD) selected the articles to be included and excluded. The search provided 1548 articles, 147 of which were duplicates. AD screened the titles and abstracts for eligibility using the free web tool RAYYAN. Of the 81 articles deemed eligible for a full reading, 51 were secondarily excluded by AD. The third (MM) and fourth (DS) authors read the remaining 30 articles and agreed to their inclusion. The authors met regularly during the selection process to clarify how the inclusion criteria were to be applied and to deliberate over the suitability of certain articles for inclusion. The selection process is presented in the PRISMA-ScR flowchart (Figure 1).

### Data Extraction

A Microsoft Excel spreadsheet was created to compile pertinent information from the selected articles. It included 15 items regarding general information (first author, year of publication, and country of publication), the methodology used (primary objective, research design, and type of data collection), the population concerned (type of recruitment, number, gender, and age of children or adolescents concerned and the number and gender of parents), and the DHI itself (technology, related distressing mental experiences, and main results). The spreadsheet was not modified during the examination of the selected articles.

### Analysis

We conducted an inductive thematic content analysis using the reflexive approach by Braun and Clake [22]. AD actively read the selected articles to obtain meaningful information and patterns in the data. He then constructed “codes” identifying the most basic and meaningful information regarding the attitudes of children, adolescents, and their parents toward DHIs. All the authors then collated these codes into categories according to their similarities; the categories were named and organized into themes showing a patterned response within the data set.

## Results

### General Information

The 30 included articles originated from 10 countries and regions: the United States (n=13, 43%), the United Kingdom (n=3, 10%), Canada (n=3, 10%), Finland (n=2, 7%), the Netherlands (n=2, 7%), Australia (n=2, 7%), Sri Lanka (n=2, 7%), Austria (n=1, 3%), New Zealand (n=1, 3%), and the European Union (n=1, 3%). The articles were published between 2010 and 2021. The number of published articles increased over time, with a peak in 2019 (Table 1).

**Table 1:** Number of articles according to year of publication (n=30).

Year of publication	Articles, n (%)
2010	2 (7)
2011	0 (0)
2012	1 (3)
2013	0 (0)
2014	2 (7)
2015	1 (3)
2016	3 (10)
2017	3 (10)
2018	4 (13)
2019	7 (23)
2020	6 (20)
2021	1 (3)

We identified 8 different ways to deliver DHIs, as mentioned by the authors of the articles included: videoconferencing (8/30, 27%), mobile apps (8/30, 27%), serious games (5/30, 17%), programs installed on a computer (1/30, 3%), electronic diaries (1/30, 3%), internet sites (2/30, 7%), mobile phones to take photos or videos (1/30, 3%), and virtual environments (1/30, 3%). However, 3 articles did not investigate a specific DHI delivery technology.

Overall, 8 different types of therapy delivered through DHIs were identified: cognitive behavioral therapy (9/30, 30%), mental health and general health education (5/30, 17%), medical examinations (5/30, 17%), group therapy (1/30, 3%), contingency management (1/30, 3%), social skills (1/30, 3%), alternative and augmentative communication (1/30, 3%), and mood monitoring (1/30, 3%). However, 6 articles did not specify the type of therapy delivered by the DHI.

The DHI discussed in various articles explored either a specific mental health problem or more general transdiagnostic symptoms. A total of 23 authors mentioned a single distressing mental experience: anxiety disorder (n=5, 17%; including anxiety in autism spectrum disorder [ASD] and social anxiety disorder), ASD (n=3, 10%), depressive disorder (n=2, 7%), substance use disorder (n=4, 13%), sleep behaviors (n=2, 7%), attention deficit with or without hyperactivity disorder (n=1, 3%), risk-taking behaviors (in terms of drugs and sexual activity; n=2, 7%), psychosis (n=1, 3%), nonsuicidal self-injury (n=1, 3%), and neurodevelopmental disorder (n=1, 3%). One article mentioned 3 distressing mental experiences (anxiety, depressive, and eating disorders), whereas another focused on mental health well-being in general. The remaining 6 articles did not mention any specific distressing mental experiences.

We identified six different places or means of recruitment from the articles as follows: (1) nonuniversity care (15/30, 50%; including local structures and general nonuniversity hospitals, general practitioners, nonuniversity mental health clinics, and mental health consultations in a specialist center); (2) schools (8/30, 27%) where consultation services were provided in middle and high schools—one article mentioned a special school for children with specific needs regarding communication skills; (3) social services (4/30, 13%; school counselors, social counselors, or community counselors); (4) community activities (2/30, 7%); (5) public advertising (2/30, 7%; posters at community events, school conferences, local conferences, and street and social media advertising); and (6) university hospitals (3/30, 10%; inpatient and outpatient consultations). Some studies (6/30, 20%) included >1 method of recruitment. One study did not indicate where the study population was recruited. Finally, the study population in one study was derived from users’ reviews published on a mobile app provider.

Overall, 4 and 5 articles recruited study populations from rural areas and underprivileged communities, respectively. Furthermore, 2 of these 9 articles were recruited from both rural and underprivileged minority communities. Underprivileged communities included low-income populations and ethnic or colored minority populations.

Of the 30 studies, 19 interviewed children and adolescents, whereas 7 interviewed children, adolescents, and their parents. Furthermore, 3 studies interviewed only parents. One study did not specify whether the data were from children, adolescents, or parents. Overall, 1891 children and adolescents were included in the study. As only 9 articles provided the mean age of the participants, we were unable to provide global statistics. Nevertheless, based on the inclusion criteria, we were able to identify different age groups: 13- to 18-year-old adolescents (22/30, 73%), 6- to 12-year-old children (9/30, 30%), 2- to 5-year-old preschool children (1/30, 3%), and 0- to 23-month-old infants (1/30, 3%). Furthermore, 7 studies included 2 different age groups.

Of the 26 studies that interviewed children and adolescents, 4 did not provide data on gender or provided incomplete data. We counted a total of 397 (24%) boys and 1215 (66%) girls in the other 22 articles. It is important to highlight that one exploratory survey included 775 teenage girls.

In total, 11 studies consulted parents on their attitudes toward the DHI used by their children and adolescents. Specifically, parents were consulted in 5 feasibility studies, 3 qualitative studies, 2 participative studies, and 1 survey. A total of 310 parents were included in this study. Only 5 studies included data on parents’ gender: among 73 parents, 60 were mothers and 13 were fathers.

### Themes of Articles

We identified 5 themes in the 30 selected articles, as defined in Textbox 1. Each article provided data on 1 to 4 of these themes (Tables 2-6).

The most frequent theme was *Use* that was found in 22 studies (Table 5) [24,28,30-37,39-43,45,47-52]: 7 exploratory studies, 8 observational studies, 3 trials, 2 randomized controlled trials, and 2 participatory research studies. Data were collected through semistructured interviews, focus groups, and surveys. These data included attendance rate, completion of intervention rate, and use rates. *Use* was investigated with regard to the current use of ICT, current use of a DHI, accessibility of the DHI evaluated in the study, and intervention completion or attendance rates. Overall, adolescents reported a wide range of uses of ICT, including entertainment, information, and social communication. Some used the internet to obtain help about problems they encountered related to their mental well-being. ICT use revealed new opportunities for care, for example, the distribution of information about drug use rehabilitation through social media to better reach adolescents and counterbalance drug cues [35]. With regard to current DHI use, an exploratory study found that 16.1% of adolescent girls with mental health problems already used a mobile app for support [39]. When evaluating a DHI, adolescents judged the aesthetic dimension, whether it was easy to use, and whether it had customizable features.

**Definitions of the 5 identified themes.**

- Satisfaction: This included measures of digital health intervention (DHI) satisfaction and acceptability as well as information on adherence to treatment.
- Use: This covered DHI intervention completion rates, attendance rates, comments about how easy or difficult the existing DHI was to use, and information on barriers or facilitators to use.
- Preferences: This regarded user preferences for different DHI treatment means and intervention features.
- Perception: This included information on the participant’s perception of the DHI used, his or her motivation to use it, and his or her perception of the DHI’s usefulness and credibility.
- Needs: This covered information on the features, content, and design of future DHIs.

**Table 2:** Characteristics of the reviewed articles (part 1).

Author	Country	Year of publication	Themes	Method	Population	Parents
Čuš et al [23]	Austria	2021	Needs, preference	Exploratory, semistructured interviews	Adolescents	No
Lopez et al [24]	United States	2020	Satisfaction, use, preference	Observational, survey, semistructured interviews	Adolescents	No
Mayworm et al [25]	United States	2020	Satisfaction, preference	Observational, survey	N/A^a^	Yes (n=125)
Hettiarachchi et al [26]	Sri Lanka	2020	Perception	Observational, semistructured interviews, focus groups	N/A	Yes (n=16)
Widnall et al [27]	United Kingdom	2020	Perception, needs, preference	Observational, focus groups	N/A	No
Newton et al [28]	Canada	2020	Needs, preference, use, satisfaction	Participatory research, surveys, semistructured interviews	Adolescents	No
Terlouw et al [29]	The Netherland	2020	Needs, preference, perception	Participatory research, focus groups, interviews	Children	Yes (n=6)
Metsäranta et al [30]	Finland	2019	Use	Trial, use rate, writes analysis	Adolescents	No
Werner-Seidler et al [31]	Australia	2019	Satisfaction, use	Trial, feasibility, survey, semistructured interviews	Adolescents	No
Quante et al [32]	United States	2019	Needs, use, preference	Observational, focus groups	Adolescents	No
Gigantesco et al [33]	Europe	2019	Use, preference	Exploratory, focus groups, semistructured interviews	Children, adolescents	No
Bagot et al [34]	United States	2019	Use, needs	Exploratory, focus groups	Adolescents	No
Soysa et al [31]	Sri Lanka	2019	Perception, needs, use	Exploratory, semistructured interviews, focus groups	N/A	Yes (n=32)
Curtis et al [35]	United States	2019	Use, perceptions	Exploratory, survey	Adolescents	No
Juárez et al [36]	United States	2018	Use, satisfaction	Observational, survey	Toddlers, preschool children	Yes (n=41)

^a^N/A: not applicable.

**Table 3:** Characteristics of the reviewed articles (part 2).

Author	Country	Year of publication	Themes	Method	Population	Parents
Carpenter et al [37]	United States	2018	Use, satisfaction	Observational, feasibility	Children and adolescents	Yes (n=11)
Clark et al [38]	Australia	2018	Perception, preference	Exploratory, semistructured interviews, focus groups	Adolescents	No
Grist et al [39]	United Kingdom	2018	Perception, use, preference	Exploratory, survey	Adolescents	No
Kong et al [40]	United States	2017	Use, satisfaction	Trial, feasibility, survey	Adolescents	No
Roberts et al [41]	Canada	2017	Satisfaction, use	Observational, survey	Adolescents	No
Hepburn et al [42]	United States	2016	Satisfaction, use	RCT^a^, feasibility, survey	Children and adolescents	Yes (n=17)
Pendergrass et al [43]	United States	2016	Perception, preference, use	Observational, semistructured interviews	Adolescents	Yes (n=8)
Sockolow et al [44]	United States	2017	Needs, preference	Participatory research, focus groups	Adolescents	No
Laine et al [45]	Finland	2016	Needs, use	Participatory research, interviews	Adolescents	Yes (n=12)
Bul et al [46]	Netherlands	2015	Perception, needs, satisfaction	Observational, survey	Children	No
Stasiak et al [47]	New Zealand	2014	Satisfaction, use	RCT, feasibility, survey, semistructured interviews	Adolescents	No
Sarver et al [48]	United States	2014	Satisfaction, use	Observational, feasibility, attendance use, survey	Children	No
Jacob et al [49]	United States	2012	Use, satisfaction	Observational, feasibility, survey	Children and adolescents	Yes (n=11)
Boydell et al [50]	Canada	2010	Perception, preference, use	Observational, semistructured interviews	Children and adolescents	No
Stallard et al [51]	England	2010	Use perceptions preferences	Exploratory, survey	Children and adolescents	Yes (n=31)

^a^RCT: randomized controlled trial.

**Table 4:** Results of the reviewed articles (part 1).

Article	Technology	Distressing mental experience	Therapy	Results
Čuš et al [23], 2021	Mobile app	NSSI^a^	CBT^b^	The intervention should offer specific features regarding NSSI; it should be customizable and suitable for adolescents.
Lopez et al [24], 2020	Videoconference	SUD^c^	Psycho or health education	Users were satisfied and the program was usable. Strength was mobility and social connection.
Mayworm et al [25], 2020	Videoconference	Not specified	Medical examination	Parents’ and users’ satisfaction was high. No difference of satisfaction between videoconference and in-person setting.
Hettiarachchi et al [26], 2020	Mobile app	Neurodevelopmental disorder	Augmentative and alternative communication	Digital interventions were perceived as mainstream technologies, accessible, as a learning tool, and challenging stigma, and concerns were raised regarding cost.
Widnall et al [27], 2020	Mobile app	Not specified	Mood monitoring	8 major themes: accessibility, flexibility, representation of mood, users' request, reflecting on mood, technical features, design, and health promotion.
Newton et al [28], 2020	Mobile app	Anxiety disorder	CBT	App should fit users’ needs and preferences, be easy to use, have relevant contents, and be aesthetic. Users’ satisfaction was high.
Terlouw et al [29], 2020	Serious game	ASD^d^	Social skills	Children’s perceptions were around 3 main topics: everyday life, social skill training, and video games. Participants helped the creation of 3 personas. Children with ASD had different goals than their parents and professionals.
Metsäranta et al [30], 2019	Electronic diary	Depressive disorder	Not specified	Half did not use the e-diary. Adolescents used it to describe their symptoms, relationships, and identity.
Werner-Seidler et al [31], 2019	Mobile app	Sleep behavior	CBT	Users were satisfied and the intervention was feasible. Users recommended several improvements. Reasons for nonadherence were documented.
Quante et al [32], 2019	Mobile app	Sleep behavior	Not specified	Adolescents were reluctant to change behavior but were open to counseling. They suggested improvement on usability and customization.
Gigantesco et al [33], 2019	Not specified	Mental health well-being	Psycho or health education	The majority preferred smartphones to communicate and used tablets at school. Collaborative games were considered more useful.
Bagot et al [34], 2019	Mobile app	SUD	Not specified	Themes were youth value rewards to reduce use, self-monitored progression, peer social support, privacy, and customization.
Soysa et al [31], 2019	Not specified	ASD	Not specified	Parents mostly used passive learning technologies. Technologies were used to teach academic skills. Future applications should fit Sri Lanka culture and be customizable.
Curtis et al [35], 2019	Not specified	SUD	Not specified	53 adolescents and 111 young adults completed the survey. Adolescents used the features of social media more. Many were exposed to drug cues on social media, whereas fewer observed recovery information. They felt that social media, smartphone apps, texting, or websites would be useful in delivering support.
Juárez et al [36], 2018	Videoconference	ASD	Medical examination	High satisfaction. Use showed that intervention saved time.

^a^NSSI: Nonsuicidal self-injury.

^b^CBT: cognitive behavioral therapy.

^c^SUD: substance use disorder.

^d^ASD: autism spectrum disorder.

**Table 5:** Results of the reviewed articles (part 2).

Author	Technology	Distressing mental experience	Therapy	Results
Carpenter et al [37], 2018	Videoconference	Anxiety disorder	CBT^a^	Intervention was feasible and acceptable. Results include barriers to care.
Clark et al [38], 2018	Internet site	Anxiety disorder	CBT	Major themes were “risks,” “efforts,” and “need for human connection.”
Grist et al [39], 2018	Mobile app	Anxiety, depressive, and eating disorder	Not specified	775 responses were gathered. 98.7% and 97.4% used the internet and apps. Only 6% used mental health mobile apps. Within those with symptoms, 15% to 17% used mental health apps. Young female adolescents massively used the internet and apps but not for mental health purposes.
Kong et al [40], 2017	Mobile phone to take photos or videos	SUD^b^	Contingency management	Intervention was feasible and acceptable. Use could be difficult.
Roberts et al [41], 2017	Videoconference	Not specified	Medical examination	High satisfaction with the use of telepsychiatry. The service was thought to be user-friendly and to help save time and money
Hepburn et al [42], 2016	Videoconference	Anxiety disorder in ASD^c^	Group therapy	Parents’ and users’ satisfaction was high. Intervention was feasible and acceptable. Users made suggestions for improvement.
Pendergrass et al [43], 2016	Serious game	Risk-taking behavior	Psycho or health education	4 themes were identified: teaching about sex, alcohol, and drugs was not done at school; a video game was a viable option; it would fit in several settings; and addition tools could be useful.
Sockolow et al [44], 2017	Serious game	Risk-taking behavior	Psycho or health education	Participants helped develop the story, reflecting their needs. Need-elicitation reflected the patient-centered care approach. Game fitted adolescents.
Laine et al [45], 2016	Internet site	Psychosis	Psycho or health education	Adolescents’ and professionals’ needs were related to the contents, usability, and design. Program was modified to satisfy adolescents’ needs.
Bul et al [46], 2015	Serious game	ADHD^d^	CBT	Game development and scientific background behind the game are described. Users were satisfied and provided reviews for further development.
Stasiak et al [47], 2014	Serious game	Depressive disorder	CBT	Intervention was feasible and acceptable. Strengths were mobility, usefulness, and that it fitted adolescents. Weakness was use difficulties.
Sarver et al [48], 2014	Virtual environments	Social anxiety disorder	CBT	Intervention was feasible, acceptable, and credible.
Jacob et al [49], 2012	Videoconference	Not specified	Medical examination	High satisfaction from parents. Telepsychiatry was thought to offer a benefit in the ability to offer medical services in underserved area.
Boydell et al [50], 2010	Videoconference	Not specified	Medical examination	4 themes arose: the encounter with the psychiatrist, the helpfulness, a sense of choice, and technology.
Stallard et al [51], 2010	Computer	Not specified	CBT	68 responses (37 young people and 31 parents) were gathered. Young people reported high level of computer use and web-based–seeking behavior. Parents were positive about computerized therapy, whereas young people expressed caution.

**Table 6:** Themes explored in the reviewed articles.

Themes	References	Sample, N	Population	Technologies	Distressing mental experiences
Use	[15-22,24,28, 30-32,34, 36,37,40, 42,45,47,48, 52]	22	Toddler Child Adolescent	Computer Electronic diary Internet site Mobile app Mobile phone (photo) Serious game Videoconference Virtual environment	Anxiety disorder in ASD^a^ Anxiety disorder Depressive disorder Eating disorder ASD Mental health well-being Psychosis Risk-taking behavior Sleep behavior Social anxiety disorder SUD^b^
Preferences	[15,16,20,33,35,37, 39-43, 50-52]	14	Child Adolescent	Mobile app Internet site Mobile app Serious game Videoconference	NSSI^c^ ADHD^d^ Anxiety disorder Depressive disorder Eating disorder ASD Risk-taking behavior Mental health well-being
Satisfaction	[15,20-22,28,30-35,45, 48]	13	Toddler Child Adolescent	Mobile app Mobile phone (photo) Serious game Videoconference Virtual environment	ADHD Anxiety disorder in ASD Anxiety disorder ASD Depressive disorder Sleep behavior Social anxiety disorder SUD
Perceptions	[18,36,37,40,42,43,47, 49-51]	11	Child Adolescent	Computer Internet site Virtual environment Mobile app Serious game Videoconference	ADHD Anxiety disorder Depressive disorder Eating disorder ASD Neurodevelopmental disorder Risk-taking behavior Social anxiety disorder SUD
Needs	[15-19,35,39, 41,43, 50]	10	Child Adolescent	Mobile app Internet site Serious game	NSSI Anxiety disorder ASD Psychosis Risk-taking behavior Sleep behavior SUD

^a^ASD: autism spectrum disorder.

^b^SUD: substance use disorder.

^c^NSSI: nonsuicidal self-injury.

^d^ADHD: attention-deficit/hyperactivity disorder.

Information on DHI user *Preferences* was found in 14 studies [23-25,27-29,32,33,38,39,43,44,46,50], specifically, 6 observational studies, 5 exploratory studies, and 3 participatory studies. Data were collected through focus groups, semistructured interviews, and surveys. Information about preferences in the results contained 2 distinct concepts. Specifically, in 8 articles, adolescents and children stated their preferences for the intervention’s or the technology’s features. In the other 6 studies, the information collected regarded user preferences for the mode of care administration: in person or via DHI. Furthermore, 3 studies found that adolescents preferred DHIs over the in-person approach, as they helped avoid possible stigma related to mental health care and reduced anxiety through teleconferencing with a psychiatrist [38,43,50]. Another 3 studies found that adolescents preferred the in-person setting to a DHI [24,39,51].

The *Satisfaction* theme appeared in 13 studies, specifically, 8 exploratory studies, 2 trials, 2 randomized controlled trials, and 1 participatory research study [24,25,28,36,37,40-42,46-49,52]. Data on satisfaction were gathered using surveys and semistructured interviews. Using Likert-scale measurements, the authors asked users or their parents whether they were satisfied with the DHI, whether they would recommend it to their friends, or whether they would use it in the future.

Information about *Perception* was found in 11 studies [26,27,29,31,35,38,39,43,50,51], specifically, 5 observational studies, 5 exploratory studies, and 1 participatory research study. Data were gathered through surveys, semistructured interviews, and focus groups. The authors measured adolescents’ and their parents’ perception of the DHI, their eagerness to use it, and their curiosity and concerns about using it. With regard to their motivation to use the DHI, some adolescents said that effort was required just as effort was required with the in-person treatment approach [38]. Others said that they were concerned about the security of the DHI that they used [34,38,39].

The least frequent theme was *Needs*, which only appeared in 10 studies [23,27-29,31,32,34,44-46], specifically 3 exploratory studies, 3 observational studies, and 4 participatory studies. Data were gathered through surveys, focus groups, and semistructured interviews. In participatory studies, elicitation of users’ needs could happen early in the DHI design process and help define the objectives of the future intervention. In one study, where professionals were also interviewed, it was found that children with ASD wanted a serious game that could help them connect with their peers, whereas professionals were more focused on social skills [29]. In general, adolescents and children expressed that DHIs should be customizable, facilitate social connection, be aesthetically pleasing, and be easy to use. These elements mirror their feedback on *Use*.

## Discussion

### Principal Findings

We conducted a rigorous scoping review exploring children’s, adolescents’, and parents’ attitudes toward DHI. We mapped and identified relevant studies, provided information on how research has been conducted to date, and presented preliminary results. After a thorough literature review, of the 1548 studies identified, 30 (1.94%) were analyzed. We extracted information related to the publication of the study, its methodology, the population concerned, and the DHI explored. We then identified 5 themes that emerged in the studies: satisfaction, use, preferences, perception, and needs.

### Population, Recruitment, and Countries of Origin

A total of 4 age groups were identified in our sample; adolescents were by far the most interviewed group. This is consistent with the fact that children and adolescents have more access to the internet and spend more time on the web as they grow and are therefore more concerned with ICT and DHI [6]. The attitudes of preschool children and infants were investigated very little, with only one study focusing on these age groups [36]. This result can be explained by the fact that there are very few DHIs designed for very young age groups [53]. For example, a scoping review published in 2019 on DHI and mental health literacy for the children aged 2 to 12 years children found only 4 results [53]. To the best of our knowledge, no previous review has explored the engagement of families of toddlers and preschool children in DHIs.

Our review focused on child and adolescent mental health service users, and therefore may have missed nonclinical users, who most probably constitute a nonnegligible part of the general population. However, the articles we analyzed came from clinical and nonclinical populations and included both DHI-naive and DHI-experienced users. It is possible that if we had not specified “mental health service users” in the search, we may have found more studies.

The wide range of study recruitment techniques described in the studies reflects the diversity of DHI uses and the services these interventions offer. This underlines the importance of the DHIs in various dimensions of care, including prevention, facilitating help-seeking behavior, self-help, and complementing face-to-face services.

Of the 30 studies, 7 (23%) specifically mentioned rural areas or underprivileged minority populations. One of the main advantages of DHIs is that they provide care to these underserved populations owing to the fact that they are accessible and cost-effective [7,54]. Only one study was conducted in a non-Western country. This underlines the insufficient research in this area in non-Western settings. It also shows a lack of DHI access in low- and middle-income countries [7,55]. This may be because of the limited resources, a shortage of skilled personnel, poor internet connectivity, and a lack of mental health policies for children and adolescents [7].

### Attitudes to DHI During the COVID-19 Pandemic

The COVID-19 pandemic crisis is seen as a pivotal point in the use of digital health in mental health. This highlights the key role that digital tools can play in terms of care when face-to-face visits are impossible [56]. However, we found no study matching our inclusion criteria that specifically explored children’s and adolescents’ attitudes toward DHIs during the COVID-19 crisis. It must be said that the time frame covered in our review (January 1, 2007, to July 8, 2021) limited the possibility to identify such articles. Some articles that we found, but were not included in the analysis, explored the transformation that mental health services underwent from providing solely in-person care before the pandemic to providing DHI during the health crisis. The authors of these studies highlighted the opportunity that the crisis provided in the field to transform health care practices [56-58].

### Preference for In-Person Interventions or DHI

We found 3 studies where adolescents and children preferred in-person interventions to DHIs [24,39,51]. Of these, 2 were exploratory studies, and therefore, participants had no hands-on experience with DHIs [39,51]. The authors of one of these 2 studies suggested that an initial meeting about DHIs with a clinician to explain the potential benefits of DHIs could improve engagement [51]. The third study concerned a mental and general health educational program delivered using teleconferencing. In that study, although adolescents found the content useful, most of them stated that they would rather prefer the program to be delivered in person to foster encounters with peers [24]. In contrast, 3 other studies highlighted that children and adolescents preferred DHIs to in-person interventions. The first DHI was a videoconference for medical examination; participants preferred it because it reduced anxiety related to meeting a mental health care professional [50]. The second was an internet site; participants preferred using it to in-person encounters, as it avoided mental health stigma [38]. The third DHI was a video game that delivered mental and general health education; participants indicated that they preferred its recreational dimension to traditional classes [43]. Logically, the preference between in-person interventions and DHIs should be explored only when the proposed service already exists in an in-person setting. In this regard, for a DHI to be the preferred choice, it should offer more features and accordingly be perceived as more useful.

### DHIs Modify the Affordance of Mental Health Care

In our review on DHIs, we gathered data showing that adolescents, children, and their parents were satisfied with the technologies used, the capabilities they offered, and their features. Many of the 30 included studies reported that DHI users were eager to use DHIs. Users sought interventions that were easy to use, flexible, customizable, and aesthetically pleasing [27]. These characteristics enhance their affordance, which is the relational structure between an object or technology and the user that enables or constrains all potential behaviors in a given context [59].

The ability to customize one’s experience when using a DHI is a valuable feature of these interventions that contributes to good affordance. For example, users can customize the interface and enable or disable geographical location [34,52]. They can also choose to install or uninstall smartphone apps. Once installed, an app may be activated in the background and consulted whenever and wherever needed to seek help at any given moment. In our review, users appreciated their ability to attend group therapy sessions from a remote location [24].

DHIs offer autonomy of use coupled with greater freedom in care seeking. Their use removes the barriers between daily living activities and engagement in care seeking.

In our review, the suitability of an intervention to children’s and adolescents’ culture was important for them. Features such as the tone of the intervention and the design of the video game characters were appreciated [29,31,44]. Suitability and usability were identified as influencing factors in the engagement of children and young people in a 2022 review by Liverpool et al [7]. The authors found that these characteristics facilitated the link between the user and the DHI and, therefore, between the user and the care delivered. Consequently, DHIs modify the affordance of mental health care.

### The Influence of Connectedness on DHI Use

In our review, an important DHI feature that adolescents sought was social connection [24,34,38]. Adolescents stated that seeking help involved other people in the same way that a feeling of connection to others provides help in itself [38]. Receiving support from other people experiencing the same type of mental health problems was helpful and came via anonymous social messaging to ensure connection and privacy [34]. Adolescents also appreciated the opportunity provided by videoconferencing to meet peers and discuss mental and general health education issues [24]. Connectedness was both a physical link enabling connection and a subjective feeling of being related to others; it was related to mental health well-being [60]. In the review by Liverpool et al [7], a sense of connectedness was identified as a major feature that DHIs should provide to ensure better engagement of adolescents and children in these types of interventions. The same authors argued that connectedness should allow people to seek support from professionals and peers in a way that allows privacy and security [7].

### Attitudes to DHIs Are Related to Trust and Fear of Stigmatization

We have observed that DHIs can increase the affordance of mental health care (ie, the understanding of the mental health care offer, its accessibility, and ease of use) and promote connectedness; the consequence of this is improved access to treatment for children and adolescents. However, increased connectedness must not be detrimental to data security and confidentiality. Some adolescents and children in our review mentioned that they were afraid of being seen with a DHI app installed on their smartphones [34]. Others were concerned about the data recorded and who might access them [27]. For some, accessing mental health care through a DHI was associated with a fear of stigmatization, linked to the worry that privacy and confidentiality could not be guaranteed using digital technology. The technologies used by DHIs must therefore be secure to facilitate engagement. In the review by Liverpool et al [7], trust and anonymity were found to promote engagement in DHIs [7].

### Implications and Recommendations

Effective use of DHIs by target audiences depends on numerous factors. In our opinion, engagement in a DHI depends on user needs and the ability of the intervention to successfully meet these needs. More specifically, the success of a DHI depends on understanding the target population’s culture and lifestyle, the difficulties that this population encounters in terms of mental health problems, and the usefulness of the intervention. DHIs should be suitable, usable, customizable, secure, and trustworthy and should facilitate connectedness. The scope and objectives of the DHI should be understandable by the user, and support should be provided throughout the duration of its use. Future research should explore the needs and specificity of the target audience. Researchers should work with the target audience and with professionals to elicit features that could be useful. This scoping review could be used as the first step of a thorough systematic literature review of children’s, adolescents’, and their parents’ attitudes toward the DHI they already use.

### Strengths and Limitations

The work described here followed established guidelines for scoping reviews [20]. We analyzed the literature and identified the themes that were studied. We considered the different types of publications, the technologies used, the distressing mental experiences studied, and the populations concerned. The results of our review provide a better understanding of the existing literature and how research in this area has been conducted. However, this review had several limitations. First, there is a risk of assessment bias because the first author performed both the study selection (ie, inclusion and exclusion) and data extraction phases alone. Second, some studies may have been missed despite our best efforts to include as many papers as possible. Third, there may have been some variation or inconsistency when grouping the results of studies under themes, as the theme definitions may partially overlap. Fourth, the variety of methodologies used in the 30 included studies made accurate comparisons difficult. Fifth, we were unable to retrieve any articles exploring the COVID-19 response that matched our inclusion criteria. This limitation could be addressed by further research. Finally, although not a limitation per se, associations between the attitudes of children and adolescents to DHIs and their use of ICT were not investigated in the articles reviewed. Familiarity with ICT could be a determining factor of DHI use and needs further investigation to better understand engagement in DHIs.

### Conclusions

The results of our scoping review show that the children and adolescents studied had a positive attitude toward DHIs. Five major themes regarding attitudes were addressed by the reviewed articles: satisfaction, preferences, use, perception, and needs. These 5 themes should be considered in future research.

We found that the experience of DHI use was a determining factor of attitudes toward these interventions. The fear of stigma in children and adolescents was associated with their propensity to use DHIs. More specifically, DHIs were perceived both as a means to avoid stigmatization and to trigger it. The connectedness of DHIs was very much sought after by adolescents and was considered a key element in mental health care. Owing to the suitability and ease of use of DHIs, participants from different studies felt that these interventions improved the affordance of mental health care.

In addition, the autonomy and freedom of use provided by DHIs erased the barriers between everyday life and engagement in seeking care. Although adolescents particularly seek connectedness, ICT allows people seeking help to reach out, maintain, and increase the connection they have with their care providers, thus modifying the healer-healed relationship.

The scalability and availability of DHIs could help reshape mental health care delivery, making it more wide-ranging and more accessible. Owing to the immediacy and transportability inherent in DHIs, the moment when and the place where mental health care is delivered can be more easily modified to best suit a patient’s lifestyle.

Finally, DHIs can help address the treatment gap in the mental health of children and adolescents, as both users and professionals can benefit from them. As the interest in DHIs grows, researchers and programmers should first investigate all the dimensions related to the target public’s attitudes toward DHIs to ensure better engagement.

## Appendix

Multimedia Appendix 1 Databases search strategy.

Multimedia Appendix 2 PRISMA-ScR (Preferred Reporting Items for Systematic reviews and Meta-Analyses extension for Scoping Reviews) checklist.

## Abbreviations

ASD: autism spectrum disorder
DHI: digital health intervention
ICT: information and communications technology
PRISMA-ScR: Preferred Reporting Items for Systematic reviews and Meta-Analyses extension for Scoping Reviews

## References

[1] Perou R, Bitsko R, Blumberg S, Pastor P, Ghandour R, Gfroerer J, Hedden SL, Crosby AE, Visser SN, Schieve LA, Parks SE, Hall JE, Brody D, Simile CM, Thompson WW, Baio J, Avenevoli S, Kogan MD, Huang LN, Centers for Disease ControlPrevention (CDC) (2013). Mental health surveillance among children--United States, 2005-2011. MMWR Suppl.

[2] Kieling C, Baker-Henningham H, Belfer M, Conti G, Ertem I, Omigbodun O, Rohde LA, Srinath S, Ulkuer N, Rahman A (2011). Child and adolescent mental health worldwide: evidence for action. Lancet.

[3] Polanczyk G, Salum G, Sugaya L, Caye A, Rohde L (2015). Annual research review: a meta-analysis of the worldwide prevalence of mental disorders in children and adolescents. J Child Psychol Psychiatry.

[4] Merikangas K, He J, Burstein M, Swanson S, Avenevoli S, Cui L, Benjet C, Georgiades K, Swendsen J (2010). Lifetime prevalence of mental disorders in U.S. adolescents: results from the National Comorbidity Survey Replication--Adolescent Supplement (NCS-A). J Am Acad Child Adolesc Psychiatry.

[5] Gulliver A, Griffiths KM, Christensen H (2010). Perceived barriers and facilitators to mental health help-seeking in young people: a systematic review. BMC Psychiatry.

[6] (2019). Children and parents: media use and attitudes report 2018. Ofcom.

[7] Liverpool S, Mota C, Sales C, Čuš A, Carletto S, Hancheva C, Sousa S, Cerón SC, Moreno-Peral P, Pietrabissa G, Moltrecht B, Ulberg R, Ferreira N, Edbrooke-Childs J (2020). Engaging children and young people in digital mental health interventions: systematic review of modes of delivery, facilitators, and barriers. J Med Internet Res.

[8] Chandrashekar P (2018). Do mental health mobile apps work: evidence and recommendations for designing high-efficacy mental health mobile apps. Mhealth.

[9] Anthes E (2016). Mental health: there's an app for that. Nature.

[10] Van Ameringen M, Turna J, Khalesi Z, Pullia K, Patterson B (2017). There is an app for that! the current state of mobile applications (apps) for DSM-5 obsessive-compulsive disorder, posttraumatic stress disorder, anxiety and mood disorders. Depress Anxiety.

[11] Larsen M, Huckvale K, Nicholas J, Torous J, Birrell L, Li E, Reda B (2019). Using science to sell apps: evaluation of mental health app store quality claims. NPJ Digit Med.

[12] Ellis L, Meulenbroeks I, Churruca K, Pomare C, Hatem S, Harrison R, Zurynski Y, Braithwaite J (2021). The application of e-mental health in response to COVID-19: scoping review and bibliometric analysis. JMIR Ment Health.

[13] Lattie E, Kornfield R, Ringland K, Zhang R, Winquist N, Reddy M (2020). Designing mental health technologies that support the social ecosystem of college students. Proc SIGCHI Conf Hum Factor Comput Syst.

[14] Haine-Schlagel R, Walsh N (2015). A review of parent participation engagement in child and family mental health treatment. Clin Child Fam Psychol Rev.

[15] Hollis C, Falconer C, Martin J, Whittington C, Stockton S, Glazebrook C, Davies EB (2017). Annual research review: digital health interventions for children and young people with mental health problems - a systematic and meta-review. J Child Psychol Psychiatry.

[16] MeSH: attitude. NCBI.

[17] Voas D (2014). Towards a sociology of attitudes. Sociol Res Online.

[18] Hawke LD, Sheikhan NY, MacCon K, Henderson J (2021). Going virtual: youth attitudes toward and experiences of virtual mental health and substance use services during the COVID-19 pandemic. BMC Health Serv Res.

[19] Faux-Nightingale A, Philp F, Chadwick D, Singh B, Pandyan A (2022). Available tools to evaluate digital health literacy and engagement with eHealth resources: a scoping review. Heliyon.

[20] Tricco AC, Lillie E, Zarin W, O'Brien KK, Colquhoun H, Levac D, Moher D, Peters MD, Horsley T, Weeks L, Hempel S, Akl EA, Chang C, McGowan J, Stewart L, Hartling L, Aldcroft A, Wilson MG, Garritty C, Lewin S, Godfrey CM, Macdonald MT, Langlois EV, Soares-Weiser K, Moriarty J, Clifford T, Tunçalp Ö, Straus SE (2018). PRISMA extension for scoping reviews (PRISMA-ScR): checklist and explanation. Ann Intern Med.

[21] d'Halluin A, Costa M, Sebbane D, Margot M Attitudes of children, adolescents, and their parents toward digital health interventions: a scoping review. JMIR Preprints..

[22] Braun V, Clarke V (2006). Using thematic analysis in psychology. Qual Res Psychol.

[23] Čuš A, Edbrooke-Childs J, Ohmann S, Plener P, Akkaya-Kalayci T (2021). "Smartphone apps are cool, but do they help me?": a qualitative interview study of adolescents' perspectives on using smartphone interventions to manage nonsuicidal self-injury. Int J Environ Res Public Health.

[24] Lopez C, Gilmore AK, Moreland A, Danielson CK, Acierno R (2020). Meeting kids where they are at-a substance use and sexual risk prevention program via telemedicine for african american girls: usability and acceptability study. J Med Internet Res.

[25] Mayworm AM, Lever N, Gloff N, Cox J, Willis K, Hoover SA (2020). School-based telepsychiatry in an urban setting: efficiency and satisfaction with care. Telemed J E Health.

[26] Hettiarachchi S, Kitnasamy G, Gopi D (2020). "Now I am a techie too" - parental perceptions of using mobile technology for communication by children with complex communication needs in the Global South. Disabil Rehabil Assist Technol.

[27] Widnall E, Grant CE, Wang T, Cross L, Velupillai S, Roberts A, Stewart R, Simonoff E, Downs J (2020). User perspectives of mood-monitoring apps available to young people: qualitative content analysis. JMIR Mhealth Uhealth.

[28] Newton A, Bagnell A, Rosychuk R, Duguay J, Wozney L, Huguet A, Henderson J, Curran J (2020). A mobile phone-based app for use during cognitive behavioral therapy for adolescents with anxiety (MindClimb): user-centered design and usability study. JMIR Mhealth Uhealth.

[29] Terlouw G, van't Veer JT, Kuipers DA, Metselaar J (2018). Context analysis, needs assessment and persona development: towards a digital game-like intervention for high functioning children with ASD to train social skills. Early Child Development Care.

[30] Metsäranta K, Kurki M, Valimaki M, Anttila M (2019). How do adolescents use electronic diaries? A mixed-methods study among adolescents with depressive symptoms. J Med Internet Res.

[31] Soysa A, Al MA (2019). Technology for children with autism spectrum disorder: what do Sri Lankan parents and practitioners want?. Interact Comput.

[32] Quante M, Khandpur N, Kontos EZ, Bakker JP, Owens JA, Redline S (2019). A qualitative assessment of the acceptability of smartphone applications for improving sleep behaviors in low-income and minority adolescents. Behav Sleep Med.

[33] Gigantesco A, Palumbo G, Zadworna-Cieślak M, Cascavilla I, Del Re D, Kossakowska K, European Group W (2019). An international study of middle school students' preferences about digital interactive education activities for promoting psychological well-being and mental health. Ann Ist Super Sanita.

[34] Bagot K, Hodgdon E, Sidhu N, Patrick K, Kelly M, Lu Y, Bath E (2019). End user-informed mobile health intervention development for adolescent cannabis use disorder: qualitative study. JMIR Mhealth Uhealth.

[35] Curtis BL, Ashford RD, Magnuson KI, Ryan-Pettes SR (2019). Comparison of smartphone ownership, social media use, and willingness to use digital interventions between generation Z and millennials in the treatment of substance use: cross-sectional questionnaire study. J Med Internet Res.

[36] Juárez AP, Weitlauf A, Nicholson A, Pasternak A, Broderick N, Hine J, Stainbrook JA, Warren Z (2018). Early identification of ASD through telemedicine: potential value for underserved populations. J Autism Dev Disord.

[37] Carpenter AL, Pincus DB, Furr JM, Comer JS (2018). Working from home: an initial pilot examination of videoconferencing-based cognitive behavioral therapy for anxious youth delivered to the home setting. Behav Ther.

[38] Clark LH, Hudson JL, Dunstan DA, Clark GI (2020). Capturing the attitudes of adolescent males’ towards computerised mental health help‐seeking. Aus Psychologist.

[39] Grist R, Cliffe B, Denne M, Croker A, Stallard P (2018). An online survey of young adolescent girls' use of the internet and smartphone apps for mental health support. BJPsych Open.

[40] Kong G, Goldberg AL, Dallery J, Krishnan-Sarin S (2017). An open-label pilot study of an intervention using mobile phones to deliver contingency management of tobacco abstinence to high school students. Exp Clin Psychopharmacol.

[41] Roberts N, Hu T, Axas N, Repetti L (2017). Child and adolescent emergency and urgent mental health delivery through telepsychiatry: 12-month prospective study. Telemed J E Health.

[42] Hepburn SL, Blakeley-Smith A, Wolff B, Reaven JA (2016). Telehealth delivery of cognitive-behavioral intervention to youth with autism spectrum disorder and anxiety: a pilot study. Autism.

[43] Pendergrass TM, Hieftje K, Crusto CA, Montanaro E, Fiellin LE (2016). If we build it, will they come? A qualitative study of key stakeholder opinions on the implementation of a videogame intervention for risk reduction in adolescents. Games Health J.

[44] Sockolow P, Schug S, Zhu J, Smith T, Senathirajah Y, Bloom S (2017). At-risk adolescents as experts in a new requirements elicitation procedure for the development of a smart phone psychoeducational trauma-informed care application. Inform Health Soc Care.

[45] Laine A, Anttila M, Välimäki M (2016). Modification of an internet-based patient education program for adults with schizophrenia spectrum disorder to suit adolescents with psychosis. Inform Health Soc Care.

[46] Bul KC, Franken IH, Van der Oord S, Kato PM, Danckaerts M, Vreeke LJ, Willems A, van Oers HJ, van den Heuvel R, van Slagmaat R, Maras A (2015). Development and user satisfaction of "Plan-it commander," a serious game for children with ADHD. Games Health J.

[47] Stasiak K, Hatcher S, Frampton C, Merry SN (2012). A pilot double blind randomized placebo controlled trial of a prototype computer-based cognitive behavioural therapy program for adolescents with symptoms of depression. Behav Cogn Psychother.

[48] Sarver NW, Beidel DC, Spitalnick JS (2014). The feasibility and acceptability of virtual environments in the treatment of childhood social anxiety disorder. J Clin Child Adolesc Psychol.

[49] Jacob MK, Larson JC, Craighead WE (2012). Establishing a telepsychiatry consultation practice in rural Georgia for primary care physicians: a feasibility report. Clin Pediatr (Phila).

[50] Boydell K, Volpe T, Pignatiello A (2010). A qualitative study of young people's perspectives on receiving psychiatric services via televideo. J Can Acad Child Adolesc Psychiatry.

[51] Stallard P, Velleman S, Richardson T (2010). Computer use and attitudes towards computerised therapy amongst young people and parents attending child and adolescent mental health services. Child Adolesc Ment Health.

[52] Werner-Seidler A, Wong Q, Johnston L, O'Dea B, Torok M, Christensen H (2019). Pilot evaluation of the Sleep Ninja: a smartphone application for adolescent insomnia symptoms. BMJ Open.

[53] Peyton D, Hiscock H, Sciberras E (2019). Do digital health interventions improve mental health literacy or help-seeking among parents of children aged 2-12 years? A scoping review. Stud Health Technol Inform.

[54] van Veen T, Binz S, Muminovic M, Chaudhry K, Rose K, Calo S, Rammal J, France J, Miller J (2019). Potential of mobile health technology to reduce health disparities in underserved communities. West J Emerg Med.

[55] Huang K, Lee D, Nakigudde J, Cheng S, Gouley KK, Mann D, Schoenthaler A, Chokshi S, Kisakye EN, Tusiime C, Mendelsohn A (2019). Use of technology to promote child behavioral health in the context of pediatric care: a scoping review and applications to low- and middle-income countries. Front Psychiatry.

[56] Torous J, Jän Myrick K, Rauseo-Ricupero N, Firth J (2020). Digital mental health and COVID-19: using technology today to accelerate the curve on access and quality tomorrow. JMIR Ment Health.

[57] Baweja R, Verma S, Pathak M, Waxmonsky JG (2020). Development of a child and adolescent tele-partial hospitalization program (tele-PHP) in response to the COVID-19 pandemic. Prim Care Companion CNS Disord.

[58] Batchelor R, Catanzano M, Kerry E, Bennett SD, Coughtrey AE, Liang H, Curry V, Heyman I, Shafran R (2020). Debate: lessons learned in lockdown - a one-day remotely delivered training on low-intensity psychological interventions for common mental health conditions. Child Adolesc Ment Health.

[59] Evans SK, Pearce KE, Vitak J, Treem JW (2016). Explicating affordances: a conceptual framework for understanding affordances in communication research. J Comput Mediat Commun.

[60] Kawachi I, Berkman L (2001). Social ties and mental health. J Urban Health.

