# Supplementary material for: Attitudes of Children, Adolescents, and Their Parents Toward Digital Health Interventions: Scoping Review
Source: J Med Internet Res. 2023 May 2;25:e43102. doi: 10.2196/43102 (PMC10189627; doi:10.2196/43102)
Supplement: Multimedia Appendix 1 [file jmir_v25i1e43102_app1.docx]

Appendix : search strategy

| MEDLINE database. From 1/01/2007 to 8/07/2021 ; age filter of 0 – 18. 1043 results found | | |
| --- | --- | --- |
| Digital mental health intervention | Child or adolescent mental health service user | Attitude |
| (("Distance Counselling"[Mesh]) OR ("Telemedicine"[Mesh]) OR ("Videoconferencing"[Mesh]) OR ("Internet-Based Intervention"[Mesh]) OR ("Computers"[Mesh]) OR ("Cell Phone"[Mesh]) OR ("Mobile Applications"[Mesh]) OR ("Biomedical Technology"[Mesh]) OR ("Software"[Mesh]) OR (”Virtual Reality Exposure Therapy”[mesh]) OR (”Virtual Reality ”[mesh] ) OR ( "Information Technology"[Mesh] ) OR "e-mental health"[tw] OR "e-mental health"[ot] OR "digital technology"[tw] OR "digital technology"[ot] OR "Digital health"[tw] OR "Digital health"[ot] OR “ e-health ”[tw] OR ” e-health “[ot] ) | AND ( “ mentally ill persons ”[mesh] OR (“ Child “[mesh] or “ adolescent ”[mesh] or patient[mesh] or user[ot] or user[tw]) AND ( ” mental Health “[mesh] OR ” psychiatry “[mesh] OR ” mental disorders “[mesh] ) ) | AND ( ( ("Motivation"[Mesh]) OR ("Attitude"[Mesh]) OR ( perspective[ot] ) OR preference[tw] OR preference[ot] OR « need* »[tw] OR “ need* “[ot] OR "expect*"[tw] OR "expect*"[ot] OR perspective[tw] OR "intention to use"[tw] OR "intention to use"[ot]) ) |
| EMBASE database, from 1/01/2007 to 8/07/2021. 131 results found.  Age filtration was obtained with the following syntax:  AND ([embryo]/lim OR [fetus]/lim OR [newborn]/lim OR [infant]/lim OR [child]/lim OR [preschool]/lim OR [school]/lim OR [adolescent]/lim) | | |
| Digital mental health intervention | Child or adolescent mental health service user | Attitude |
| ( ’e-counseling’/exp OR 'telemedicine’/exp OR 'telehealth'/exp OR 'video consultation'/exp OR 'videoconferencing'/exp OR 'web-based intervention'/exp OR 'computer'/exp OR 'mobile phone'/exp OR 'mobile application'/exp OR 'software'/exp OR 'virtual reality exposure therapy'/exp OR 'virtual reality'/exp OR 'information technology'/exp OR 'digital technology'/exp OR 'digital health' ) | AND ( ( 'mental patient'/exp ) OR ( ('mental health'/exp OR 'mental health service'/exp OR 'psychiatry'/exp OR 'mental disease'/exp) AND ('patient'/exp or user.mp OR child*.mp or adolescen*.mp) ) ) | AND ('motivation'/exp OR 'attitude'/exp OR perspective OR prefer*.mp OR need.mp OR expect*.mp OR 'satisfaction'/exp) |
| PsycINFO database, from 1/01/2007 to 8/07/2021, with general filter: “peer-reviewed journal only”, excluding “memoir” and “book”  Two separate searches were performed using different age filters:  Childhood (birth – 12 years): 148 results found. Adolescence (13-17 years): 226 results found. | | |
| Digital mental health intervention | Child or adolescent mental health service user | Attitude |
| ( DE "Telemedicine" OR DE "Online Therapy" OR DE "Teleconferencing" OR DE "Teleconsultation" OR DE "Telepsychiatry" OR DE "Telepsychology" OR DE "Telerehabilitation" OR DE "Electronic Health Services" OR DE "Digital Interventions" OR DE « Mobile » OR DE "Computer Assisted Therapy" OR DE "Internet" OR DE "Online Therapy" OR DE "Computer Applications" OR DE "Computer Assisted Therapy" OR DE "Computer Applications" OR DE "Computer Simulation" OR DE "Computer Software" OR DE "Mobile Applications" OR DE "Computers" OR DE "Computer Peripheral Devices" OR DE "Microcomputers" OR DE "Mobile Devices" OR DE "Mobile Phones" OR DE "Smartphones" OR DE "Text Messaging" OR DE "Mobile Devices" OR DE "Mobile Phones" OR DE "Tablet Computers" OR DE "Mobile Health" OR DE "Mobile Technology" OR DE "Wearable Devices" OR DE "Virtual Reality" OR DE "Augmented Reality" OR DE "Computer Simulation" OR DE "Virtual Reality Exposure Therapy" OR DE "Information and Communication Technology" OR DE "Digital Technology" OR DE "Health Information Technology" OR DE "Digital Interventions" ) | AND ( ( DE "Psychiatric Patients" OR DE « clients » ) OR ( ( DE "Patients" OR DE "Hospitalized Patients" OR DE "Medical Patients" OR DE "Outpatients" OR TX ” user ” OR TX “ child* ” OR TX “adolesce* ” ) AND ( DE "Mental Health" OR DE "Mental Status" OR DE "Mental Health Services" OR DE "Community Mental Health Services" OR DE "Psychiatry" OR DE "Adolescent Psychiatry" OR DE "Biological Psychiatry" OR DE "Child Psychiatry" OR DE "Community Psychiatry" OR DE "Consultation Liaison Psychiatry" OR DE "Forensic Psychiatry" OR DE "Geriatric Psychiatry" OR DE "Military Psychiatry" OR DE "Neuropsychiatry" OR DE "Orthopsychiatry" OR DE "Social Psychiatry" OR DE "Transcultural Psychiatry" OR DE "Mental Disorders" OR DE "Affective Disorders" OR DE "Anxiety Disorders" OR DE "Autism Spectrum Disorders" OR DE "Bipolar Disorder" OR DE "Borderline States" OR DE "Chronic Mental Illness" OR DE "Dissociative Disorders" OR DE "Eating Disorders" OR DE "Gender Dysphoria" OR DE "Mental Disorders due to General Medical Conditions" OR DE "Neurocognitive Disorders" OR DE "Neurodevelopmental Disorders" OR DE "Neurosis" OR DE "Paraphilias" OR DE "Personality Disorders" OR DE "Psychosis" OR DE "Serious Mental Illness" OR DE "Sleep Wake Disorders" OR DE "Somatoform Disorders" OR DE "Stress and Trauma Related Disorders" OR DE "Substance Related and Addictive Disorders" OR DE "Thought Disturbances" ) ) ) | AND ( TX "Computer Attitudes" OR TX "Computer Usage" OR TX "Internet Usage" OR TX "Smartphone Use" OR TX "Motivation" OR TX "Needs" OR TX "Attitudes" OR TX "Preferences" OR TX "Expectations" OR TX "Client Attitudes" OR TX "Client Satisfaction" OR MM "Needs Assessment" ) |
